# ^18^F-THK5351 PET imaging in patients with progressive supranuclear palsy: associations with core domains and diagnostic certainty

**DOI:** 10.1038/s41598-020-76339-0

**Published:** 2020-11-10

**Authors:** Jung-Lung Hsu, Shih-Hsin Chen, Ing-Tsung Hsiao, Chin-Song Lu, Tzu-Chen Yen, Nobuyuki Okamura, Kun-Ju Lin, Yi-Hsin Weng

**Affiliations:** 1 Department of Neurology, New Taipei Municipal TuCheng Hospital, New Taipei City, Taiwan; 2 Department of Neurology, Chang Gung Memorial Hospital, Linkou Medical Center, No. 5, Fuxing St., Guishan, Taoyuan, Taiwan; 3 College of Medicine, Neuroscience Research Center, Chang Gung University, Taoyuan, Taiwan; 4 Graduate Institute of Mind, Brain, and Consciousness, Taipei Medical University, Taipei, Taiwan; 5 Brain and Consciousness Research Center, TMU Shuang Ho Hospital, New Taipei City, Taiwan; 6 Department of Nuclear Medicine and Center for Advanced Molecular Imaging and Translation, Chang Gung Memorial Hospital, Linkou Medical Center, No. 5, Fuxing St., Guishan, Taoyuan, Taiwan; 7 Healthy Aging Research Center and Department of Medical Imaging and Radiological Sciences, College of Medicine, Chang Gung University, Taoyuan, Taiwan; 8 Neuroscience Research Center, Chang Gung Memorial Hospital, Linkou Medical Center, Taoyuan, Taiwan; 9 APRINOIA Therapeutics Inc., Taipei, Taiwan; 10 Department of Pharmacology, Faculty of Medicine, Tohoku University, Sendai, Japan; 11 Tohoku Medical and Pharmaceutical University, Sendai, Japan; 12 Department of Nuclear Medicine, Keelung Chang Gung Memorial Hospital, Keelung, Taiwan; 13 Neurological Clinic, Taoyuan, Taiwan

**Keywords:** Movement disorders, Neurodegeneration

## Abstract

The associations of ^18^F-THK5351 tau positron emission tomography (PET) findings with core domains of progressive supranuclear palsy (PSP) and its diagnostic certainty have yet to be fully elucidated. The ^18^F-THK5351 PET patterns of 17 patients with PSP (68.9 ± 6.5 years; 8 women) were compared with those observed in 28 age-matched and sex-matched (66.2 ± 4.5 years, 18 women) control subjects (CS). Tracer accumulation—as reflected by standardized uptake value ratios (SUVRs) and z-scores—was correlated with core domains of PSP and different levels of diagnostic certainty. Compared with CS, patients with PSP showed an increased ^18^F-THK5351 uptake in the globus pallidus and red nucleus. Patients with PSP and oculomotor dysfunction had significantly higher SUVRs in the midbrain, red nucleus, and raphe nucleus than those without. In addition, cases who meet criteria for level 1 (highest) certainty in the postural instability domain showed significantly higher SUVRs in the frontal, parietal, precuneus, and sensory-motor cortex. Patients with probable PSP had significantly higher SUVR values than those with possible PSP in multiple cortical (i.e., frontal, parietal, temporal, anterior cingulate gyrus, precuneus, and sensory-motor gyrus) and subcortical (i.e., putamen, thalamus, and raphe nucleus) regions. Patterns of ^18^F-THK5351 uptake were correlated to core domains of PSP—including oculomotor dysfunction and postural instability. Moreover, the degree of diagnostic certainty for PSP was appreciably associated with ^18^F-THK5351 PET findings.

## Introduction

Progressive supranuclear palsy (PSP) is a severe progressive atypical parkinsonian syndrome characterized by supranuclear gaze palsy, axial rigidity, postural instability, and cognitive dysfunction^1^. The neuropathological hallmark of the disease consists in the intra-neuronal aggregation of four-repeat tau fibrils in the subthalamic nucleus, globus pallidus, substantia nigra, red nucleus, oculomotor nucleus, and cortical regions^2–4^.

PSP remains diagnostically challenging as patients with typical signs and symptoms can have different underlying pathologies; conversely, cases who share identical neuropathological findings may differ in terms of clinical presentations^5,6^. Ultimately, the definite diagnosis of PSP requires post-mortem neuropathological examination. In a recent milestone, the International Parkinson and Movement Disorder Society (MDS) has outlined clinical diagnostic criteria for PSP and its four core domains (oculomotor dysfunction, postural instability, akinesia, and cognitive dysfunction). In doing so, different degrees of diagnostic certainty were proposed—resulting in sensitivity and specificity values > 85% in a neuropathologically-defined cohort^7,8^. The application of the new MDS criteria is expected to facilitate the clinical diagnosis of classic (PSP–Richardson syndrome) and variant presentations of PSP in a more accurate fashion^7,9^. Notably, four different clinical subtypes of PSP can be distinguished based on the presence of various core domains^10^.

Neuroimaging findings may be useful to support the clinical diagnosis of PSP. The anteroposterior diameter of midbrain and the midbrain-to-pons ratio measured on magnetic resonance (MR) imaging are recognized imaging biomarkers of classic PSP^11^. Positron emission tomography (PET) can also play a role in excluding or confirming the diagnosis of PSP when ^18^F-fluorodeoxyglucose (FDG) or dopamine-based tracers are used^12–14^. Moreover, tau PET ligands (e.g., ^18^F-AV-1451 and ^18^F-THK5351)—which can trace tau fibrils in the brain—have been investigated in patients with PSP^15^. An enhanced ^18^F-AV-1451 uptake has been reported in the putamen and globus pallidus of patients with PSP and has been shown to correlate with the PSP rating scale^16,17^. However, other studies failed to demonstrate an association between ^18^F-AV-1451 accumulation and measures of motor dysfunction or longitudinal changes in the PSP rating scale ^18,19^. An autopsy study also reported that tau neuropathology in the basal ganglia correlated with FDG—but not ^18^F-AV-1451—uptake^20^. The early development of tau-tracer is suffering from the off-target binding to monoamine oxidase B (MAO-B)^21,22^. Nonetheless, astrogliosis-related MAO-B elevation is a common histopathological known feature of all parkinsonian syndromes, and there is consistent evidence that accumulation of ^18^F-THK5351 in PET images obtained from living patients correlates with symptom severity and post-mortem tau pathology in PSP brains^23–25^.

The associations of ^18^F-THK5351 PET findings with core domains of PSP and its diagnostic certainty have yet to be fully elucidated. We set out to examine this issue in the current study of patients with PSP (diagnosed according to the new MDS criteria) and age- and sex-matched control subjects (CS).

## Materials and methods

### Study population

We examined 17 patients with PSP (68.9 ± 6.5 years; 8 women) and 28 age-matched and sex-matched CS (66.2 ± 4.5 years, 18 women). All cases were recruited from tertiary medical centers and had PSP diagnosed according to the MSD criteria. Patients were excluded if they were taking monoamine oxidase inhibitors (MAOI). Demographic characteristics, age at disease onset, and time from diagnosis to PET imaging were obtained from all participants. The degrees of diagnostic certainty for PSP in general (i.e., definite PSP, probable PSP, possible PSP, and suggestive of PSP) and for each of the four core domains were assigned according to the MDS criteria. Control subjects had a Mini-Mental State Examination score above the optimal cut-off according to their age and education^26^. Participants were excluded if they had a history of stroke, head trauma, or alcoholism. All procedures complied with the tenets of the 1964 Helsinki Declaration and its later amendments. All participants gave written informed consent and the study protocol was approved by the Institutional Review Board of the Chang Gung Memorial Hospital (CGMHIRB No. 201601674A0). The study was registed in *clinicaltrials.gov* (NCT03386669) on December 21, 2017.

### Image acquisition

^18^F-THK5351—which was synthesized and prepared at the cyclotron facility of the Chang Gung Memorial Hospital^27^—was administed intravenously (378 ± 17 MBq) and patients rested confortably for 60 min. PET/CT was performed on a Siemens Biograph mCT 16 scanner (Siemens, Erlangen, Germany) and images were reconstructed using the three-dimensional ordered subset-expectation maximization algorithm (24 subsets and 4 iterations; Gaussian filter = 2 mm; zoom = 3). A CT scan was performed for attenuation correction, along with scatter and random corrections. Reconstructed images had a matrix size of 400 × 400 × 148 (voxel size, 0.68 × 0.68 × 1.5 mm^3^)^28^. Brain MR was performed on a 3-T scanner (Magneton Trio, Siemens) with the following sequences: (1) axial fluid attenuation inversion recovery (FLAIR) (TR = 9000 ms, TE = 87 ms, T1 = 2500 ms, voxel size = 0.9 × 0.7 × 4 mm^3^); (2) axial three-dimensional (3D) high resolution T1-weighted magnetization prepared rapid acquisition gradient echo (MP-RAGE) (TR = 2000 ms, TE = 2.63 ms, T1-900 ms, flip angle = 9°, voxel size = 1 × 1 × 1 mm^3^); and (3) coronal T2-weighted turbo spin echo (TR = 7,400 ms, TE = 95 ms, voxel size = 0.4 × 0.4 × 2 mm^3^) perpendicularly to the long hippocampal axis.

### Image analysis

All imaging data were transformed for further processing into the Neuroimaging Informatics Technology Initiative (NIFTI) format using the PMOD image analysis software (version 3.7; PMOD Technologies Ltd., Zurich, Switzerland). Standardized uptake value images from ^18^F-THK5351 PET and T1-weighted MR images in the native space were analyzed in parallel for each participant. Anatomical coregistration was performed by analyzing PET and MR images simultaneously using the SPM12 toolbox (https://www.fil.ion.ucl.ac.uk/spm/software/spm12) as previously described^29,30^. ^18^F-THK5351 PET and MR images were carefully matched using anatomical landmarks, and the Muller-Gartner method was used for partial volume correction^31^. Native high-resolution MR images were normalized to the Montreal Neurological Institute (MNI) standard space using the normalization toolbox provided in SPM12^32^. The transform matrix was subsequently applied to PET images. The mean intensity across the cerebellar gray matter was used as the reference region for calculation of standardized uptake value ratios (SUVRs) from ^18^F-THK5351 PET images^24^. Fifteen regions of interest (ROIs) were selected according to the published literature and the Harvard–Oxford cortical structural atlas^18,24,33^. Bilateral cortical (i.e., frontal, parietal, temporal, occipital lobes, sensory-motor cortex, anterior cingulate gyrus, and precuneus) and subcortical (i.e., caudate, putamen, globus pallidus, thalamus, red nucleus, midbrain, raphe nucleus, and dentate nucleus) regions were included. SUVRs were assessed for each ROI. Means and standard deviations for SUVRs were determined from PET images of CS. For each participant, z-transformed images were calculated according to the following formula: (individual SUVR image—mean SUVR image)/standard deviation of SUVR image. Mean z-values for each ROI were derived from z-transformed ^18^F-THK5351 PET SUVR images. MR images were analyzed in the native space on the mid-sagittal view. Upon measurements of midbrain and pons diameters, the midbrain-to-pons ratio (midbrain diameter/pons diameter) was calculated as described previously^34^.

### Statistical analysis

Summary statistics for continuous variables were presented as mean ± standard deviation and 95% confidence intervals, and comparisons between groups were performed using ANOVA with Bonferroni multiple comparison. Categorical data were summarized as frequencies and percentages, and differences between groups were analyzed with the chi-squared test. Adjustment for age and sex with linear regression was applied when regional SUVR values were compared. When core domains were analyzed, postural instability (n = 16) and akinesia (n = 17) were common, whereas oculomotor (n = 8) and cognitive (n = 2) dysfunction occurred more rarely. Owing to their high frequency, a subgroup analysis of the two first domains was performed based on the degree of diagnostic certainty, as follows: postural instability, P1 group (n = 8) versus non-P1 group (n = 8); akinesia, A1 group (n = 9) versus non-A1 group (n = 6). When a core domain occurred rarely (i.e., oculomotor and cognitive dysfunction), the degree of certainty was considered higher^8^ and subgroup analyses were not implemented^8^. Linear regression was used to analyze associations between MR parameters and ^18^F-THK5351 PET imaging variables in patients with PSP. All analyses were untaken using SPSS, version 21.0 (IBM, Armonk, NY, USA). Two-tailed p values < 0.05 were considered statistically significant.

## Results

### Sample characteristics

The general characteristics of the study participants are presented in Table 1. Patients with PSP were of similar age (p = 0.17) and sex (p = 0.26) as CS. The mean time interval from disease onset to imaging for patients with PSP was 6.6 ± 3.8 years. There were no significant correlations between disease duration and midbrain-to-pons ratios on MR or SUVRs from ^18^F-THK5351 PET. The results for core domains of PSP and diagnostic certainty are shown in Table 2. Postural instability and akinesia (as noted above) were commonly observed, whereas oculomotor and cognitive dysfunction were rare. The following degrees of diagnostic certainty for PSP were identified: probable PSP (n = 7), possible PSP (n = 5), and suggestive of PSP (n = 5).

**Table 1:** General characteristics of patients with PSP and control subjects.

	Patients with PSP (n = 17)	Control subjects (n = 28)	p
Mean age, years	68.9 ± 6.5	66.2 ± 4.5	0.17
Men/women	9/8	10/18	0.26
Education, years	11.01 ± 4.7	10.9 ± 4.5	0.89
Age at onset, years	62.9 ± 7.1	–	NA
Interval from disease onset to imaging, years	6.6 ± 3.8	–	NA
MMSE	–	27.1 ± 1.7	NA

*PSP* progressive supranuclear palsy, *NA* not available, *MMSE* Mini-Mental State Examination. Data represent the mean (± standard deviation, SD).

**Table 2:** Demographics, PSP core domains, diagnostic certainty, and MR measurements in patients with PSP diagnosed with the new MDS criteria.

Case #	Age, years	Sex	PSP core domains				Diagnostic certainty	Phenotype	MR measurements		
			Oculomotor dysfunction	Postural instability	Akinesia	Cognitive dysfunction			Midbrain, mm	Pons, mm	Midbrain-to-pons ratio
S002	63	M	–	2	3	–	Suggestive	PSP-P	9.90	15.59	0.64
S004	73	F	2	1	2	–	Probable	PSP-RS	7.39	14.71	0.50
S005	66	F	–	2	3	–	Suggestive	PSP-P	9.40	16.07	0.58
S008	64	F	–	2	1	–	Suggestive	PSP-PGF	8.67	15.80	0.55
S013	79	M	1	2	1	–	Probable	PSP-PGF	8.62	16.65	0.52
S014	68	M	1	1	2	1	Probable	PSP-RS	9.43	16.75	0.56
S025	63	F	1	3	1	–	Probable	PSP-PGF	9.57	15.65	0.61
S029	67	M	–	1	1	–	Possible	PSP-PGF	10.8	18.06	0.59
S033	71	M	–	2	1	–	Possible	PSP-PGF	8.83	15.07	0.59
S036	61	M	3	2	1	–	Possible	PSP-PGF	9.70	17.34	0.56
S037	60	M	–	2	1	–	Possible	PSP-PGF	10.5	18.16	0.58
S039	68	M	1	1	–	2	Probable	PSP-RS	8.91	18.23	0.49
S040	82	F	2	1	1	–	Probable	PSP-RS	10.87	16.90	0.64
S041	79	F	–	1	2	–	Suggestive	PSP-P	10.11	17.30	0.58
S042	71	M	–	–	1	–	Possible	PSP-PGF	9.65	16.54	0.58
S043	70	F	–	1	–	–	Suggestive	PSP-PI	11.12	17.31	0.64
S044	74	F	2	1	2	–	Probable	PSP-RS	9.60	18.40	0.52

– absent, *PSP* progressive supranuclear palsy, *MR* magnetic resonance, *MDS* International Parkinson and Movement Disorder Society, *M* male, *F* female, *PSP-P* progressive supranuclear palsy with predominant parkinsonism, *PSP-RS* progressive supranuclear palsy-Richardson syndrome, *PSP-PI* progressive supranuclear palsy with predominant postural instability, *PSP-PGF* progressive supranuclear palsy with progressive gait freezing.

### Regional mean SUVRs differences

**Figure 1:**
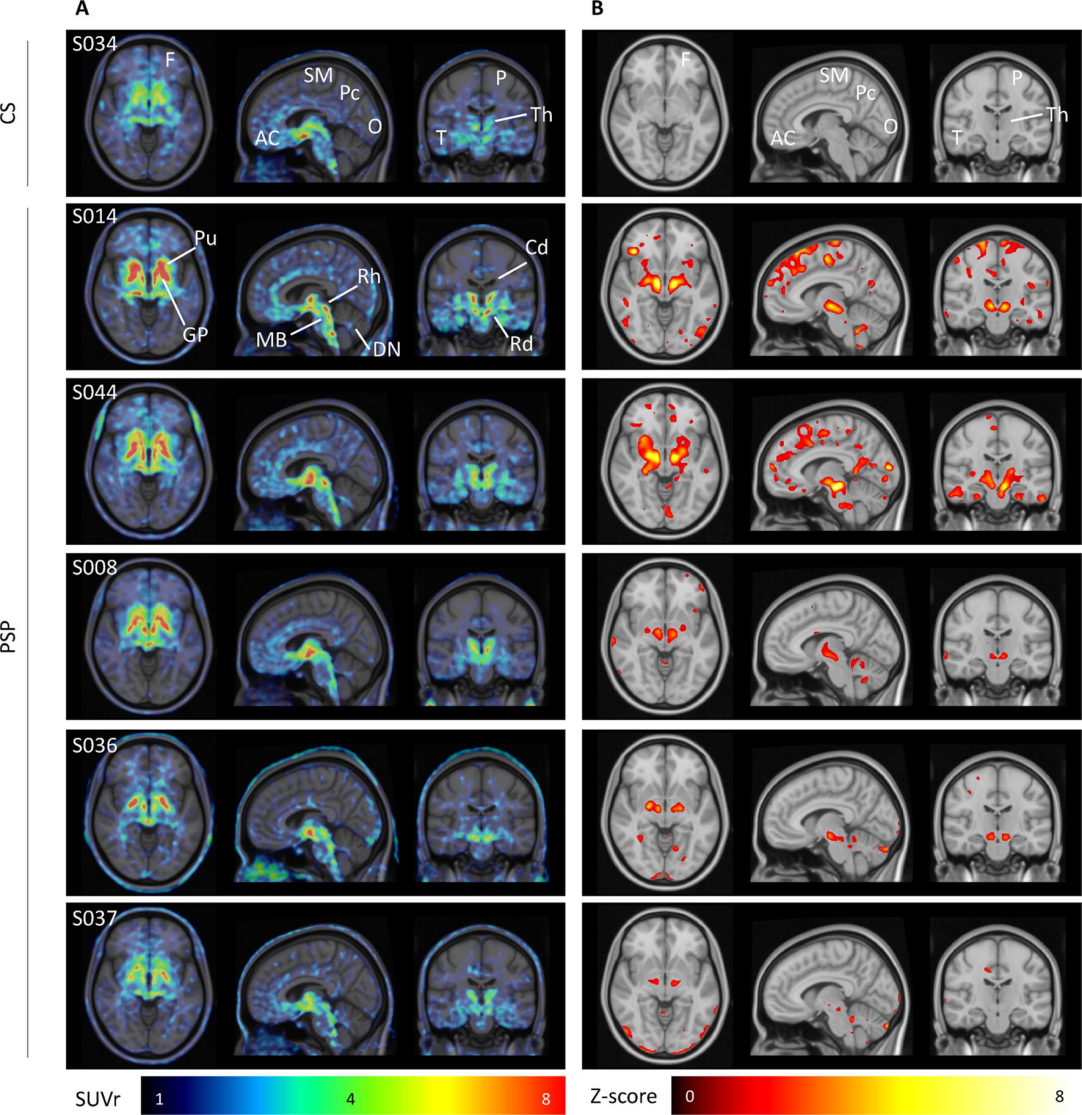
Representative ^18^F-THK5351 SUVR images (**A**) and corresponding Z-score images (**B**) in one control subject (CS) and five patients with progressive supranuclear palsy (PSP). The z-score images highlight the voxel-based comparisons of each PSP subject (n = 17) to all CS (n = 28). Only voxels with z-score values above 1.96 (95% confidence interval) were considered. (*AC *anterior cingulate, *Cd* caudate nucleus, *DN* dentate nucleus, *F* frontal, *GP* globus pallidus, *MB* midbrain, *O* occipital, *P* parietal, *Pc* precuneus, *Pu* putamen, *T* temporal, *Rd* red nucleus, *Rh* raphe nucleus, *SM* Sensory-motor gyrus, *Th* thalamus).

**Figure 2:**
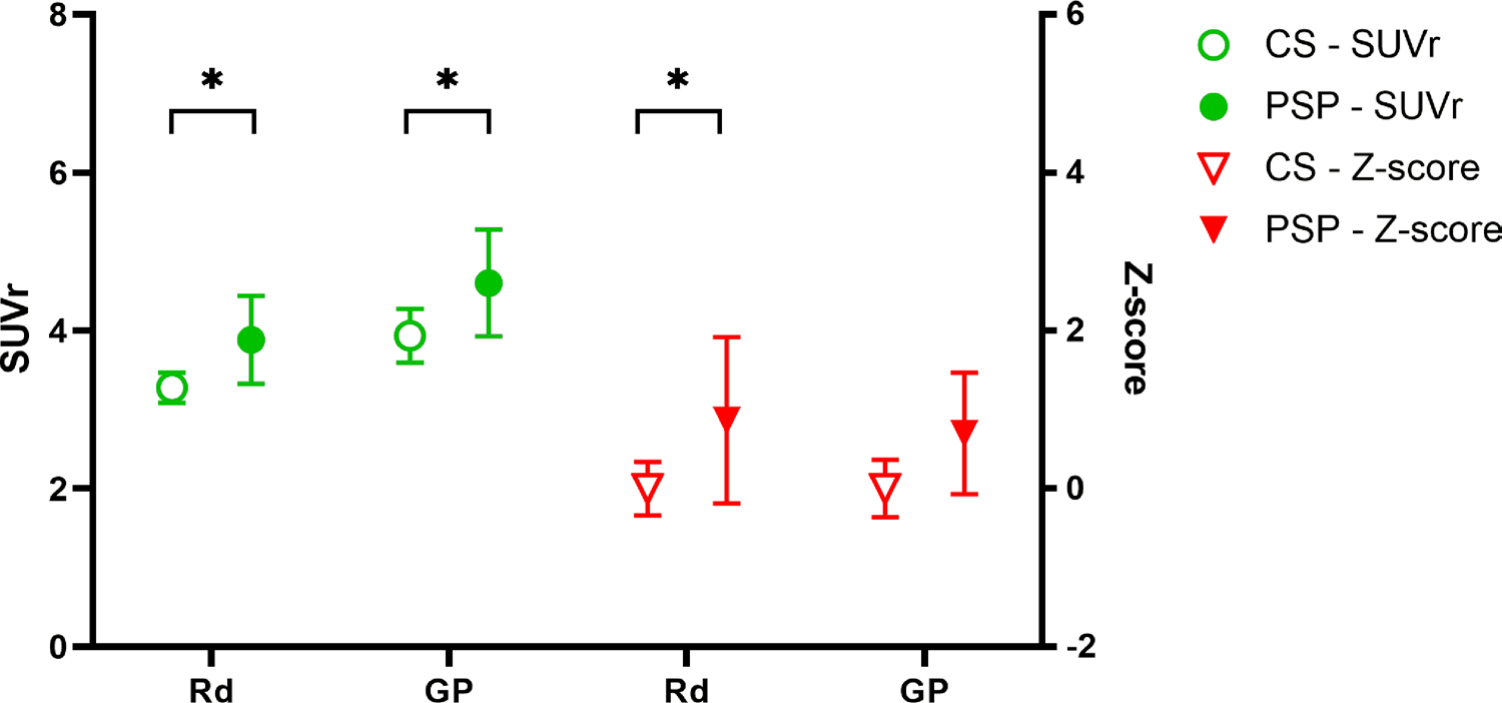
Regional ^18^F-THK5351 standard uptake value ratio (SUVR) and Z-score of red nucleus (Rd) and globus pallidus (GP) in patient with progressive supranuclear palsy (PSP, n = 17) and control subjects (CS, n = 28). Data present in mean with 95% confidence interval. (ANOVA with Bonerroni multiple comparison *p < 0.05).

**Figure 3:**
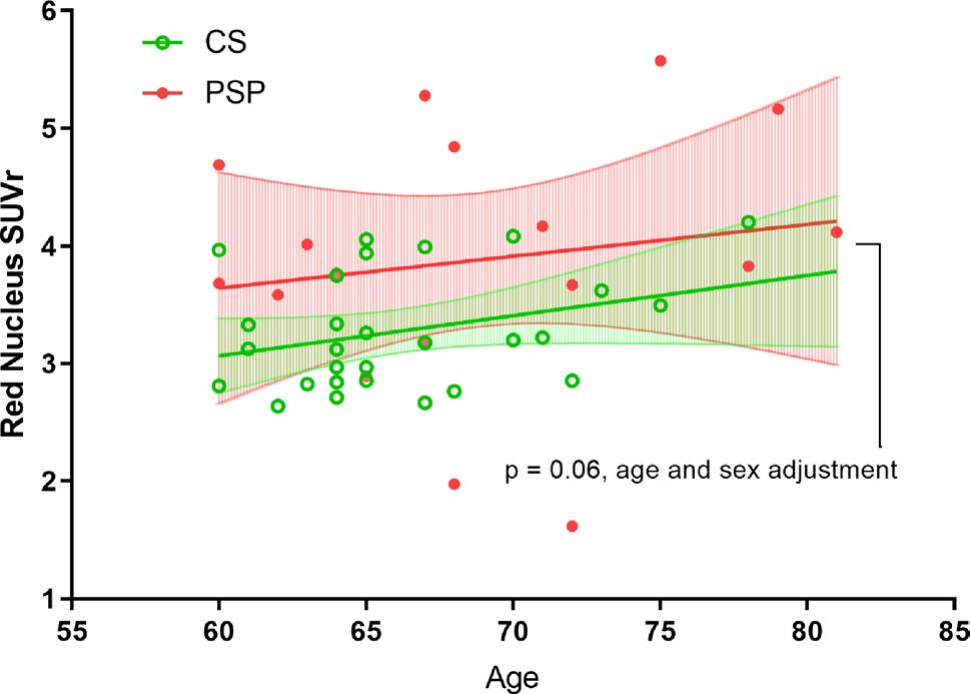
Red nucleus ^18^F-THK5351 standard uptake value ratio (SUVR) in patient with progressive supranuclear palsy (PSP, n = 17) and control subjects (CS, n = 28). Data present in mean with 95% confidence interval. (p = 0.06 after adjustment for age and sex).

Representative ^18^F-THK5351 PET images obtained from a patients with PSP and a CS are shown in Fig. 1. We found no intergroup differences in regional mean SUVRs values measured in the frontal, parietal, temporal, and occipital cortex, as well as in the precuneus and anterior cingulate gyrus (Table 3). As for subcortical regions, patients with PSP showed an increased ^18^F-THK5351 accumulation in the globus pallidus and red nucleus compared with CS (all p < 0.05). More details regarding to left and right hemisphere measurements are shown in Supplementary Table 1. Similar results were observed for z-transformed ^18^F-THK5351 PET SUVR images (Fig. 2). ^18^F-THK5351 uptake was significantly lower in the caudate and thalamus of patients with PSP compared with CS (all p < 0.05). The impact of age and sex on ^18^F-THK5351 accumulation was investigated in the entire study sample. Men had significantly higher mean SUVR values (3.79 ± 0.89) in the red nucleus than women (3.29 ± 0.69, p = 0.01). Age was positively associated with ^18^F-THK5351 SUVR values in the putamen (p = 0.0003), globus pallidus (p = 0.001), and dentate nucleus (p = 0.02). Adjustment for age and sex revealed no statistically significant association between an increased ^18^F-THK5351 accumulation in the red nucleus in patients with PSP compared with CS (p = 0.06; Fig. 3). Conversely, there was a lower tracer uptake in the caudate (p = 0.0008) and thalamus (p = 0.001) of patients with PSP. The low uptake possibly due to radioactivity underestimate by spill-in from ventricle in PSP subjects with severe brain atrophy even after partial volume correction.

**Table 3:** Differences in regional SUVR values between patients with PSP and control subjects.

Region	Patients with PSP (n = 17)	Control subjects (n = 28)	PSP–CS (95% CI of difference)	p
Frontal cortex	1.29 ± 0.29	1.31 ± 0.17	− 0.560 to 0.56	ns
Parietal cortex	1.14 ± 0.22	1.19 ± 0.17	− 0.63 to 0.52	ns
Temporal cortex	1.52 ± 0.38	1.64 ± 0.19	− 0.68 to 0.47	ns
Occipital cortex	1.11 ± 0.15	1.11 ± 0.15	− 0.58 to 0.58	ns
Sensory-motor gyrus	1.04 ± 0.16	1.04 ± 0.17	− 0.58 to 0.58	ns
Precuneus	1.38 ± 0.28	1.44 ± 0.20	− 0.64 to 0.52	ns
Anterior cingulated gyrus	2.03 ± 0.51	2.17 ± 0.32	− 0.72 to 0.44	ns
Thalamus	2.77 ± 0.92	3.42 ± 0.53	− 1.22 to − 0.07	0.0159
Caudate nucleus	1.71 ± 0.87	2.59 ± 0.66	− 1.46 to − 0.31	0.0001
Putamen	3.56 ± 1.22	3.39 ± 0.67	− 0.41 to 0.74	ns
Midbrain	3.11 ± 0.48	3.09 ± 0.48	− 0.77 to 0.38	ns
Red nucleus	3.88 ± 1.08	3.28 ± 0.49	0.03 to 1.18	0.0312
Globus pallidus	4.60 ± 1.32	3.93 ± 0.88	0.09 to 1.24	0.0106
Raphe nucleus	5.11 ± 1.86	4.93 ± 0.73	− 0.39 to 0.77	ns
Dentate nucleus	1.88 ± 0.33	1.93 ± 0.19	− 0.62 to 0.54	ns

ANOVA with Bonferroni’s multiple comparisons test. Data represent the mean (± standard deviation, SD).

*SUVR* standardized uptake value ratio, *PSP* progressive supranuclear palsy, *ns* not significant, *CI* confidence interval.

### ^18^F-THK5351 PET findings, core domains of PSP, and diagnostic certainty

**Figure 4:**
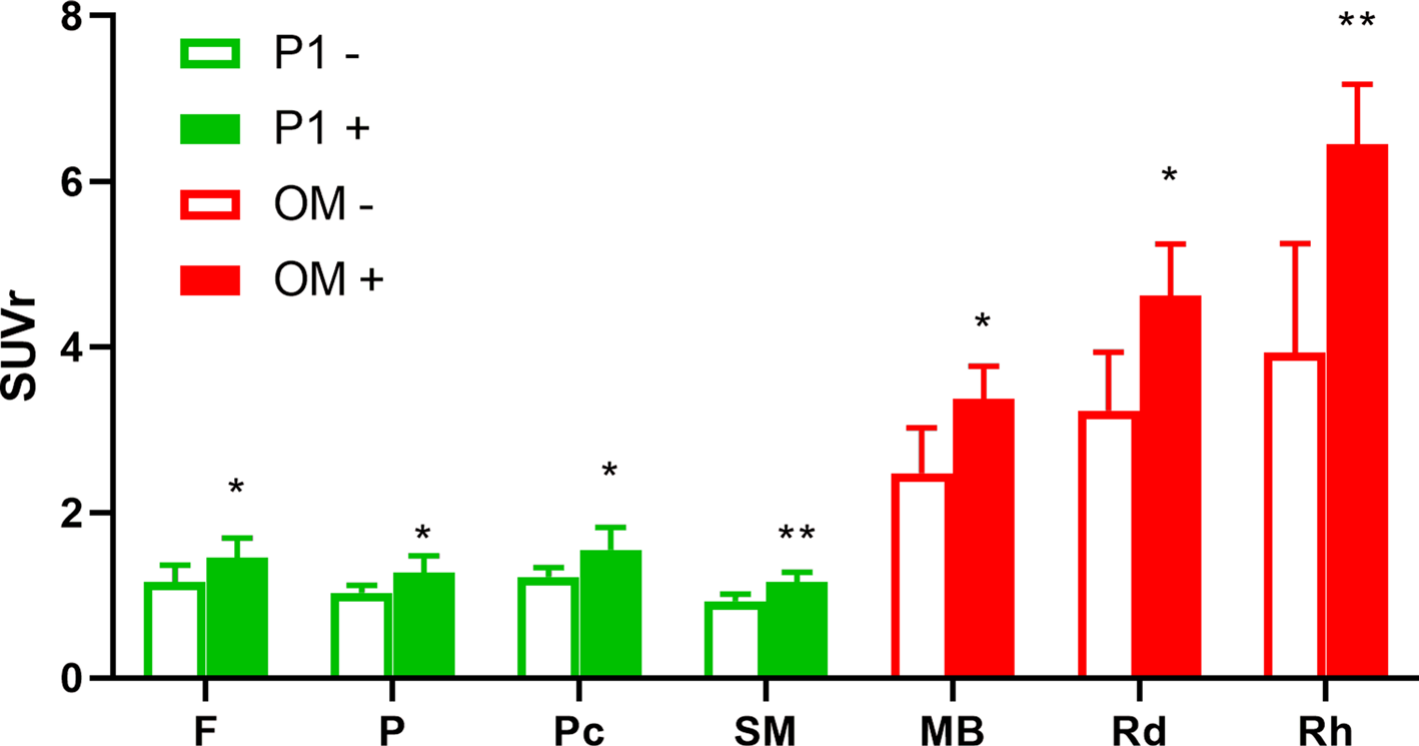
Association between regional ^18^F-THK5351 standard uptake value ratio (SUVR) and progressive supranuclear palsy core domains of postural instability (P1) and oculomotor dysfunction (OM). Data present in mean with 95% confidence interval. (*F* frontal, *P* parietal, *Pc* precuneus, *SM* sensory-motor gyrus).

Patients with ocolomotor dysfunction had significantly higher SUVR values (3.37 ± 0.47) in the midbrain region compared with those without (2.47 ± 0.72, p = 0.008, Fig. 4). Similar higher values were observed in the red nucleus (present versus absent: 4.61 ± 0.75 versus 3.23 ± 0.91, respectively, p = 0.02) and in the raphe nucleus (present versus absent: 6.45 ± 0.86 versus 3.93 ± 1.71, respectively, p = 0.002; Fig. 4). Patients who meet level 1 certainty criteria for the postural instability domain showed significantly higher SUVRs in the frontal (p = 0.04), parietal (p = 0.03), precuneus (p = 0.02) and sensory-motor cortex (p = 0.003; Fig. 4). However, no significant differences were observed for subcortical regions. ^18^F-THK5351 uptake in cortical and subcortical regions did not differ significantly with respect to the akinesia and cognitive dysfunction domains. As for diagnostic certainty, SUVR values were significantly higher in patients with probable PSP than in those with possible PSP in multiple cortical regions—including frontal (p = 0.03), parietal (p = 0.009), and temporal (p = 0.01) cortex, as well as in the anterior cingulate gyrus (p = 0.009), precuneus (p = 0.03) and sensorimotor gyrus (p = 0.02). Similar findings were observed for the following subcortical regions: putamen (p = 0.02), thalamus (p = 0.01), and raphe nucleus (p = 0.02).

### Relations between MR findings, PSP core domains, and levels of diagnostic certainty

The MRI analysis between patients with PSP and CS showed a significantly smaller midbrain diameter (PSP vs CS = 9.59 ± 0.19 vs 10.86 ± 0.15, p < 0.01) and a smaller midbrain-to-pons ratio (PSP vs CS = 0.57 ± 0.01 vs 0.63 ± 0.01, p < 0.01). The is no significant between group difference in pons diameters (p = 0.13). There was no significant difference in the midbrain-to-pons ratio for patients with and without ocolomotor dysfunction (p = 0.08). This variable was unrelated to postural instability (P1 versus non-P1, p = 0.83), akinesia (A1 versus non-A1, p = 0.56), and cognitive dysfunction (present versus absent, p = 0.13). Neither diagnostic certainty nor different clinical phenotypes of PSP showed significant associations with midbrain diameters or midbrain-to-pons ratios.

### Relations between midbrain-to-pons ratio and regional SUVR values

**Figure 5:**
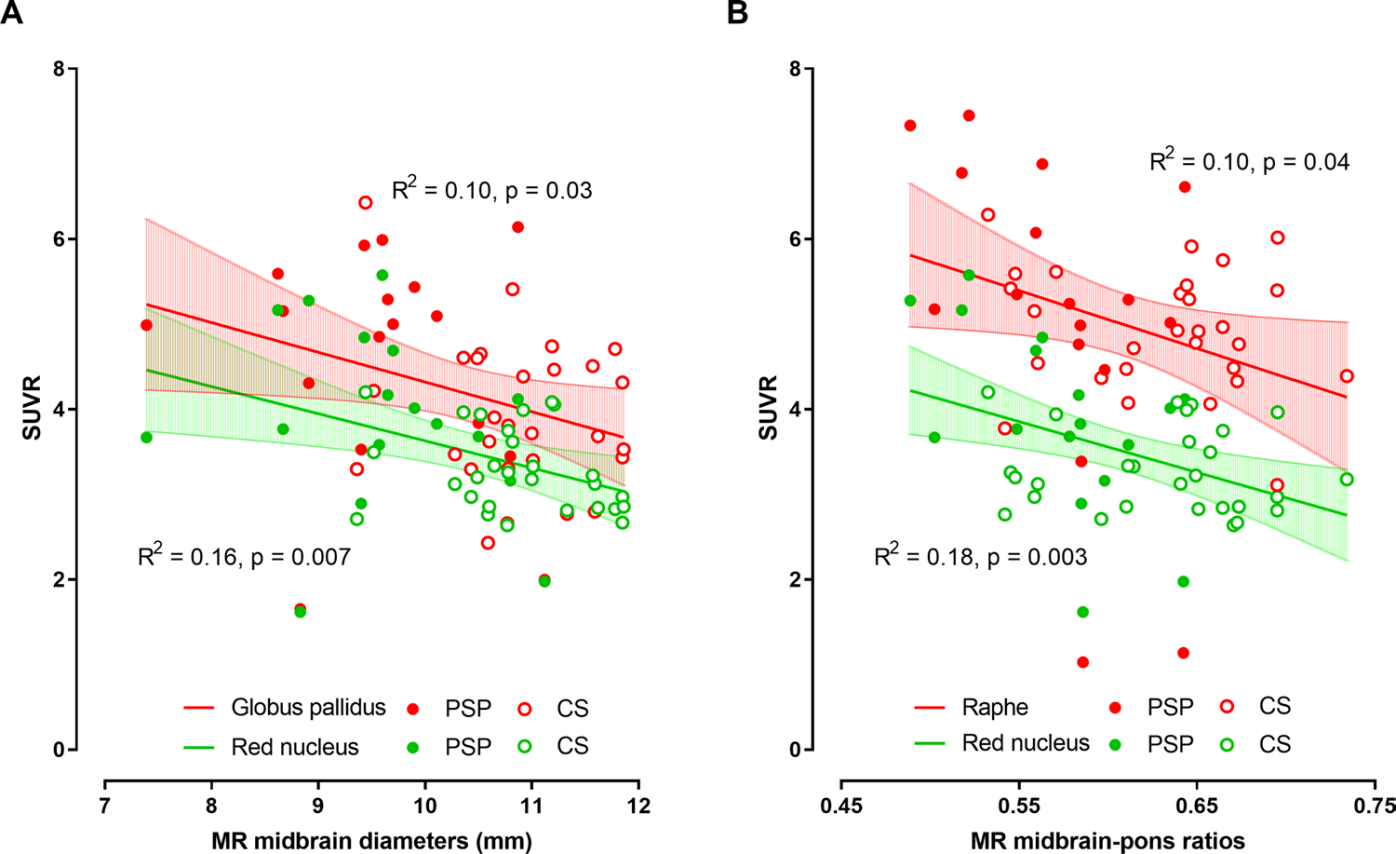
Correlation between regional ^18^F-THK5351 SUVR and MRI midbrain diameter (**A**) and midbrain-to-pons ratio (**B**). Data present in mean with 95% confidence interval.

The MRI midbrain diameter showed a negative correlation with SUVR values in the red nucleus and globus pallidus (Fig. 5A). Similarly, the MRI midbrain-to-pons ratio was inversely related to SUVR values in the red nucleus and raphe (Fig. 5B). These associations persisted even after adjustment for age and sex in red nucleus (p = 0.030 and p = 0.033, respectively).

## Discussion

There are six main findings from our study, as follows: (1) SUVR values in the globus pallidus and red nucleus from ^18^F-THK5351 PET imaging can differentiate between patients with PSP and CS; (2) patients with PSP and oculomotor dysfunction have significantly higher SUVR values in the midbrain, red nucleus, and raphe nucleus than those without; (3) patients with PSP who meet level 1 certainty criteria for the postural instability domain show significantly higher SUVRs in the frontal, parietal, precuneus, and sensory-motor gyrus; (4) SUVR values in multiple cortical and subcorticol regions are significantly higher in patients with probable PSP than in those with possible PSP; (5) regional SUVRs values measured in the red nucleus and midbrain regions are inversely related to the midbrain-to-pons ratio measured on MRI; (6) neither diagnostic certainty nor clinical phenotypes of PSP are significantly associated with the midbrain-to-pons ratio.

### Diagnostic criteria and tau PET imaging in patients with PSP

Several PET studies have used first-generation tau ligands (e.g., ^18^F-AV1451) to examine the patterns of tracer uptake in relation to the clinical features of PSP diagnosed according to the new MSD criteria^17,35^. In general, patients with PSP are characterized by an increased ^18^F-AV1451 accumulation in the midbrain, basal ganglia, and dentate nuclear regions; however, similar patterns do not seem disease-specific as they have been observed in CS of similar age^16^. Moreover, subcortical tracer accumulation in PSP is not related to clinical symptoms^18^. Of note, pathological studies have shown that ^18^F-AV1451 binds with relatively low affinity to tau deposits—frequently in the same range of CS^36,37^. Differently from ^18^F-AV1451, ^18^F-THK5351 is characterized by a higher binding selectivity to tau deposition occurring in PSP brains^23,25^. Moreover, ^18^F-THK5351 PET findings have been shown to correlate with clinical symptoms and severity in patients with PSP diagnosed according to the National Institute of Neurological Disorders and Stroke and the Society for PSP (NINDS-SPSP) criteria^9,24^.

To our knowledge, this study is the first to describe ^18^F-THK5351 PET findings in patients with PSP who were diagnosed according to the new MDS criteria. Interestingly, both SUVR values and z-scores in the globus pallidus and red nucleus were able to distinguish patients with PSP from CS. It is therefore possible that tau accumulation in these areas as assessed by ^18^F-THK5351 PET imaging may have diagnostic utility in PSP. However, interpretation of PET results obtained with first-generation tau tracers can be made difficult by off-target tracer binding to neuromelanin and/or hemorrhagic lesions, as well as by the use of MAOI^23,38^. Although ^18^F-THK5351 PET and MR images were carefully matched, our results should be interpreted cautiously and require confirmation in future independent studies using second-generation tau ligands—including MK-6240, ^18^F-JNJ64349311, and ^18^F-APN1607^39,40^.

### ^18^F-THK5351 PET findings and PSP core domains

The new MDS criteria for PSP diagnosis set out four core disease domains and four distinct levels of diagnostic certainty. The combination of different core domains allows identifying distinct PSP subtypes. As for diagnostic certainty, low scores reflect a greater diagnostic confidence^8^. A previous MR study investigated the imaging correlates of oculomotor dysfunction and postural instability^41^. The results revealed that alterations in the midbrain region and the superior cerebellar peduncle were associated with vertical oculomotor dysfunction and postural instability, respectively. The relation between midbrain damage and oculomotor dysfunction has been subsequently confirmed in independent studies^42,43^. Our ^18^F-THK5351 PET findings indicate that oculomotor dysfunction is associated with higher SUVR values in the midbrain, red nucleus, and raphe nucleus—a result in accordance with published MR studies. A further confirmatory observation is the inverse correlation between the midbrain-to-pons ratio and SUVR values in the raphe nuclear and red nuclear within the midbrain region. Nonetheless, the midbrain-to-pons ratio per se showed no association with the presence of oculomotor dysfunction. However, we found that patients with PSP who met level 1 certainty criteria for the postural instability domain showed significantly higher SUVRs in the frontal, parietal, precuneus, and sensory-motor gyrus—suggesting an increased tau accumulation in these areas. A previous MR investigation demonstrated that atrophy of the superior cerebellar peduncle may be an imaging feature of early postural instability in PSP−Richardson’s syndrome^41^. Other studies have identified microstructural damage to the corpus callosum and thalamic dysfunction as potential substrates for postural instability in patients with PSP^44,45^. To our knowledge, there are no previous investigations of tau imaging in relation to PSP-associated postural instability. However, post-mortem findings from a patient with PSP and predominant postural instability revealed an increased tau accumulation in the frontal and parietal lobes^46^—a finding in accordance with our current data. Herein we show that ^18^F-THK5351 PET imaging may increase the diagnostic sensitivity for postural instability in PSP from 37 to 51%, ultimately indicating the clinical utility of this imaging modality.

### ^18^F-THK5351 PET findings and PSP diagnostic certainty

The present study is the first to correlate ^18^F-THK5351 PET findings with different levels of diagnostic certainty for PSP. Our data indicate that patients with probable PSP had significantly higher SUVR values than those with possible PSP in multiple cortical (i.e., frontal, parietal, temporal, anterior cingulate gyrus, precuneus, and sensory-motor gyrus) and subcortical (putamen, thalamus, and raphe nucleus) regions—a result consistent with published neuropathology findings^4^. These data suggest that a higher burden of tau accumulation corresponds to a greater degree of diagnostic certainty. Albeit preliminary, our results may prompt additional investigations into the role of ^18^F-THK5351 PET as an imaging tool to increase the diagnostic certainty of PSP.

### Limitations

This study has several potential weaknesses. We recognize that neuropathological assessment of the post-mortem brain is required to reach a definitive diagnosis of PSP. In the small sample examined, most patients had PSP–Richardson’s syndrome and no general conclusions can be drawn for other PSP subtypes. Moreover, ^18^F-THK5351 is an early-generation tau ligand that may exhibit off-target binding^15^. In this context, it is possible that areas of increased tracer uptake might not simply reflect tau deposition, but the presence of astrogliosis (either with or without other neuropathological alterations)^47^. Finally, image coregistration and correction for partial volume effects were based on a complex approach. However, we cannot rule out the presence of residual confounding deriving from the presence of atrophy in specific areas (e.g., caudate and thalamus). Other spatial transformation tools may help refine our approach in future studies^48^.

## Conclusion

Patterns of ^18^F-THK5351 uptake were correlated to core domains of PSP—including oculomotor dysfunction and postural instability. Moreover, the degree of diagnostic certainty for PSP was appreciably associated with ^18^F-THK5351 PET findings.

## Supplementary information

Supplementary Table 1.
