# Author Correction: ^18^F-THK5351 PET imaging in patients with progressive supranuclear palsy: associations with core domains and diagnostic certainty

**DOI:** 10.1038/s41598-021-83535-z

**Published:** 2021-02-09

**Authors:** Jung-Lung Hsu, Shih-Hsin Chen, Ing-Tsung Hsiao, Chin-Song Lu, Tzu-Chen Yen, Nobuyuki Okamura, Kun-Ju Lin, Yi-Hsin Weng

**Affiliations:** 1 Department of Neurology, New Taipei Municipal TuCheng Hospital, New Taipei City, Taiwan; 2 Department of Neurology, Chang Gung Memorial Hospital, Linkou Medical Center, No. 5, Fuxing St., Guishan, Taoyuan Taiwan; 3 College of Medicine, Chang Gung University, Taoyuan, Taiwan; 4 Graduate Institute of Mind, Brain, and Consciousness, Taipei Medical University, Taipei, Taiwan; 5 Brain and Consciousness Research Center, TMU Shuang Ho Hospital, New Taipei City, Taiwan; 6 Department of Nuclear Medicine and Center for Advanced Molecular Imaging and Translation, Chang Gung Memorial Hospital, Linkou Medical Center, No. 5, Fuxing St., Guishan, Taoyuan Taiwan; 7 Healthy Aging Research Center and Department of Medical Imaging and Radiological Sciences, College of Medicine, Chang Gung University, Taoyuan, Taiwan; 8 Neuroscience Research Center, Chang Gung Memorial Hospital, Linkou Medical Center, Taoyuan, Taiwan; 9 APRINOIA Therapeutics Inc., Taipei, Taiwan; 10 Department of Pharmacology, Faculty of Medicine, Tohoku University, Sendai, Japan; 11 Tohoku Medical and Pharmaceutical University, Sendai, Japan; 12 Department of Nuclear Medicine, Keelung Chang Gung Memorial Hospital, Keelung, Taiwan; 13 Neurological Clinic, Taoyuan, Taiwan

Correction to: *Scientific Reports* https://doi.org/10.1038/s41598-020-76339-0, published online 10 November 2020

The original version of this Article contained errors. Jung-Lung Hsu and Yi-Hsin Weng were incorrectly affiliated with ‘College of Medicine, Neuroscience Research Center, Chang Gung University, Taoyuan, Taiwan’. The correct affiliation is listed below:

College of Medicine, Chang Gung University, Taoyuan, Taiwan

In addition, the Acknowledgements section in this Article was incomplete.

“The authors acknowledge the administrative support of the Chang Gung Memorial Hospital Clinical Trial Center—which is funded by the Taiwanese Ministry of Health and Welfare (Grants MOHW104-TDU-B-212-113003, MOHW105-TDU-B-212-13302, and MOHW106-TDU-B-212-113005). This study was financially supported by grants from the Taiwanese Ministry of Health and Welfare (MOHW107-TDU-B-212-123005, MOHW108-TDU-B-212-133005), Ministry of Science and Technology (MOST 106-2314-B-182A-026-MY3, MOST 106-2314-B-182-017-MY3), and the Chang Gung Memorial Hospital Research Fund (CMRPG3F2181, CORPG3J0342, BMRPD69, CMRPG3J0371, CMRPD1H0391-3, BMRP488).”

now reads:

“The authors acknowledge the administrative support of the Chang Gung Memorial Hospital Clinical Trial Center—which is funded by the Taiwanese Ministry of Health and Welfare (grants MOHW104-TDU-B-212-113003, MOHW105-TDU-B-212-13302, and MOHW106-TDU-B-212-113005). This study was financially supported by grants from the Taiwanese Ministry of Health and Welfare (MOHW107-TDU-B-212-123005, MOHW108-TDU-B-212-133005), Ministry of Science and Technology (MOST 106-2314-B-182A-026-MY3, MOST 106-2314-B-182-017-MY3), and the Chang Gung Memorial Hospital Research Fund (CMRPG3F2181, CORPG3J0342, BMRPD69, CMRPG3D1293, CMRPG3J0371, CMRPD1H0391-3, BMRP488).”

These errors have now been corrected in the PDF and HTML versions of the Article.

