# Supplementary material for: 18F-THK5351 PET imaging in patients with progressive supranuclear palsy: associations with core domains and diagnostic certainty
Source: Sci Rep. 2020 Nov 10;10:19410. doi: 10.1038/s41598-020-76339-0 (PMC7656245; doi:10.1038/s41598-020-76339-0)
Supplement: Supplementary file 1 — Supplementary Table 1. [file 41598_2020_76339_MOESM1_ESM.doc]

**^18^F-THK5351 PET imaging in patients with progressive supranuclear palsy: associations with core domains and diagnostic certainty**

Jung-Lung Hsu^1,2,3,4†^, Shih-Hsin Chen^5†^, Ing-Tsung Hsiao^5,6^, Chin-Song Lu^7^, Tzu-Chen Yen^5,8^, Nobuyuki Okamura^9,10^, Kun-Ju Lin^5,6*^, Yi-Hsin Weng^2,3,6,7*^

^1^ Department of Neurology, New Taipei Municipal TuCheng Hospital, New Taipei City, Taiwan

^2^ Department of Neurology, Chang Gung Memorial Hospital, Linkou Medical Center, Taoyuan, Taiwan

^3^ College of Medicine, Neuroscience Research Center, Chang Gung University, Taoyuan, Taiwan

^4^ Graduate Institute of Mind, Brain, and Consciousness, Taipei Medical University, Taipei and Brain and Consciousness Research Center, TMU Shuang Ho Hospital, New Taipei City, Taiwan

^5^ Department of Nuclear Medicine and Center for Advanced Molecular Imaging and Translation, Chang Gung Memorial Hospital, Linkou Medical Center, Taoyuan, Taiwan

^6^ Healthy Aging Research Center and Department of Medical Imaging and Radiological Sciences, College of Medicine, Chang Gung University, Taoyuan, Taiwan

^7^ Neuroscience Research Center, Chang Gung Memorial Hospital, Linkou Medical Center, Taoyuan, Taiwan

^8^ APRINOIA Therapeutics Inc., Taipei, Taiwan

^9^ Tohoku University and Department of Pharmacology, Faculty of Medicine, Sendai, Japan

^10^ Tohoku Medical and Pharmaceutical University, Sendai, Japan

† Jung-Lung Hsu and Shih-Hsin Chen contributed equally to this work.

Supplement Table 1

Differences in regional SUVR values between patients with PSP and control subjects

| Region |  | Patients with PSP  (n = 17) | Control subjects  (n = 28) | PSP – CS  (95% CI of diff.) | P |
| --- | --- | --- | --- | --- | --- |
| Frontal cortex | R | 1.33 ± 0.17 | 1.31 ± 0.29 | 0.57 to -0.61 | ns |
|  | L | 1.29 ± 0.17 | 1.28 ± 0.30 | 0.58 to -0.61 | ns |
| Parietal cortex | R | 1.22 ± 0.17 | 1.17 ± 0.23 | 0.54 to -0.65 | ns |
|  | L | 1.17 ± 0.18 | 1.12 ± 0.21 | 0.53 to -0.65 | ns |
| Temporal cortex | R | 1.65 ± 0.20 | 1.55 ± 0.38 | 0.49 to -0.69 | ns |
|  | L | 1.62 ± 0.18 | 1.51 ± 0.39 | 0.48 to -0.70 | ns |
| Occipital cortex | R | 1.14 ± 0.14 | 1.14 ± 0.15 | 0.59 to -0.59 | ns |
|  | L | 1.08 ± 0.15 | 1.08 ± 0.15 | 0.59 to -0.59 | ns |
| Sensory-motor gyrus | R | 1.08 ± 0.19 | 1.05 ± 0.17 | 0.56 to -0.63 | ns |
|  | L | 1.00 ± 0.15 | 1.03 ± 0.16 | 0.62 to -0.56 | ns |
| Precuneus | R | 1.43 ± 0.20 | 1.36 ± 0.28 | 0.52 to -0.66 | ns |
|  | L | 1.46 ± 0.21 | 1.41 ± 0.29 | 0.54 to -0.64 | ns |
| Anterior cingulated gyrus | R | 2.16 ± 0.32 | 2.02 ± 0.53 | 0.45 to -0.74 | ns |
|  | L | 2.18 ± 0.33 | 2.04 ± 0.49 | 0.45 to -0.73 | ns |
| Thalamus | R | 3.41 ± 0.52 | 2.74 ± 0.93 | -0.07 to -1.25 | 0.0135 |
|  | L | 3.43 ± 0.55 | 2.80 ± 0.92 | -0.03 to -1.22 | 0.0267 |
| Caudate nucleus | R | 2.56 ± 0.67 | 1.62 ± 0.84 | -0.35 to -1.53 | <0.0001 |
|  | L | 2.64 ± 0.69 | 1.81 ± 0.92 | -0.23 to -1.42 | 0.0004 |
| Putamen | R | 3.46 ± 0.70 | 3.61 ± 1.26 | 0.75 to -0.44 | ns |
|  | L | 3.33 ± 0.65 | 3.51 ± 1.20 | 0.77 to -0.42 | ns |
| Midbrain |  | 3.09 ± 0.48 | 2.90 ± 0.75 | 0.40 to -0.79 | ns |
| Red nucleus | R | 3.18 ± 0.50 | 3.74 ± 0.97 | 1.15 to -0.03 | ns |
|  | L | 3.37 ± 0.52 | 4.02 ± 1.21 | 1.24 to 0.06 | 0.0167 |
| Globus pallidus | R | 3.86 ± 0.83 | 4.59 ± 1.33 | 1.32 to 0.14 | 0.0034 |
|  | L | 4.01 ± 0.94 | 4.62 ± 1.31 | 1.20 to 0.01 | 0.0407 |
| Raphe nucleus |  | 4.93 ± 0.73 | 5.11 ± 1.86 | 0.78 to -0.40 | ns |
| Dentate nucleus |  | 1.93 ± 0.20 | 1.89 ± 0.33 | 0.55 to -0.63 | ns |

ANOVA with Bonferroni’s multiple comparisons test. Data represent the mean (± standard deviation, SD). Abbreviations: SUVR, standardized uptake value ratio; PSP, progressive supranuclear palsy; ns: not significant; CI: confidence interval.
